# Eccentric Ergometer Training Promotes Locomotor Muscle Strength but Not Mitochondrial Adaptation in Patients with Severe Chronic Obstructive Pulmonary Disease

**DOI:** 10.3389/fphys.2017.00114

**Published:** 2017-03-03

**Authors:** Norah J. MacMillan, Sophia Kapchinsky, Yana Konokhova, Gilles Gouspillou, Riany de Sousa Sena, R Thomas Jagoe, Jacinthe Baril, Tamara E. Carver, Ross E. Andersen, Ruddy Richard, Hélène Perrault, Jean Bourbeau, Russell T. Hepple, Tanja Taivassalo

**Affiliations:** ^1^Department of Kinesiology, McGill UniversityMontreal, QC, Canada; ^2^Respiratory Epidemiology and Clinical Research Unit, McGill University Health CenterMontreal, QC, Canada; ^3^Département de Sciences de l'activité Physique, Faculté des Sciences, Université du Québec À Montréal, Complexe des SciencesMontreal, QC, Canada; ^4^Pulmonary Division, Jewish General Hospital, McGill UniversityMontreal, QC, Canada; ^5^Department of Sport Medicine and Functional Explorations, Centre Hospitalier Universitaire de Clermont-FerrandClermont-Ferrand, France; ^6^Department of Critical Care Medicine, McGill University Health CenterMontreal, QC, Canada

**Keywords:** endurance exercise training, COPD, muscle dysfunction, rehabilitation, hypertrophy, cross sectional area, mitochondrial biogenesis, respiration

## Abstract

Eccentric ergometer training (EET) is increasingly being proposed as a therapeutic strategy to improve skeletal muscle strength in various cardiorespiratory diseases, due to the principle that lengthening muscle actions lead to high force-generating capacity at low cardiopulmonary load. One clinical population that may particularly benefit from this strategy is chronic obstructive pulmonary disease (COPD), as ventilatory constraints and locomotor muscle dysfunction often limit efficacy of conventional exercise rehabilitation in patients with severe disease. While the feasibility of EET for COPD has been established, the nature and extent of adaptation within COPD muscle is unknown. The aim of this study was therefore to characterize the locomotor muscle adaptations to EET in patients with severe COPD, and compare them with adaptations gained through conventional concentric ergometer training (CET). Male patients were randomized to either EET (*n* = 8) or CET (*n* = 7) for 10 weeks and matched for heart rate intensity. EET patients trained on average at a workload that was three times that of CET, at a lower perception of leg fatigue and dyspnea. EET led to increases in isometric peak strength and relative thigh mass (*p* < 0.01) whereas CET had no such effect. However, EET did not result in fiber hypertrophy, as morphometric analysis of muscle biopsies showed no increase in mean fiber cross-sectional area (*p* = 0.82), with variability in the direction and magnitude of fiber-type responses (20% increase in Type 1, *p* = 0.18; 4% decrease in Type 2a, *p* = 0.37) compared to CET (26% increase in Type 1, *p* = 0.04; 15% increase in Type 2a, *p* = 0.09). EET had no impact on mitochondrial adaptation, as revealed by lack of change in markers of mitochondrial biogenesis, content and respiration, which contrasted to improvements (*p* < 0.05) within CET muscle. While future study is needed to more definitively determine the effects of EET on fiber hypertrophy and associated underlying molecular signaling pathways in COPD locomotor muscle, our findings promote the implementation of this strategy to improve muscle strength. Furthermore, contrasting mitochondrial adaptations suggest evaluation of a sequential paradigm of eccentric followed by concentric cycling as a means of augmenting the training response and attenuating skeletal muscle dysfunction in patients with advanced COPD.

## Introduction

Locomotor muscle dysfunction is a highly prevalent and disabling condition associated with chronic obstructive pulmonary disease (COPD), and is characterized by significant weakness and increased fatigability. The structural and metabolic alterations that underlie these symptoms in patients with COPD include muscle atrophy and erosion of oxidative capacity, which is reflected by low mitochondrial content and a fiber-type shift toward a glycolytic phenotype (Gosker et al., 2007b; Picard et al., 2008). Of important note, patients who develop these muscle alterations have a further exacerbation of exercise intolerance, a reduced health-related quality of life and pre-mature mortality compared to patients with the same pulmonary disease severity but normal muscle function (Marquis et al., 2002; Maltais et al., 2014). Therefore, strategies that target skeletal muscle in COPD will have a clinically meaningful impact on mobility and health status.

One increasingly proposed therapeutic strategy targeting skeletal muscle weakness in cardiorespiratory disease involves eccentric ergometer training (EET). Eccentric exercise takes advantage of unique properties of lengthening muscle actions that lead to a higher force-generating capacity compared to concentric, shortening actions (Enoka, 1996; Hortobagyi et al., 1996; LaStayo et al., 2003). Moreover, an additional advantage of eccentric muscle actions is that they require a lower metabolic cost and cardiopulmonary load for a given level of force production. As such, it has been shown that at the same metabolic demand, eccentric exercise allows for a four- to five-fold higher muscle mechanical load than conventional concentric exercise (Lindstedt et al., 2001; Meyer et al., 2003; Isner-Horobeti et al., 2013). Because the intensity of muscle contraction is a principal determinant of muscle growth and strength development, moderate load eccentric-based cycling protocols are being applied at relatively low cardiovascular demands in both healthy aging and cardiac disease populations, and shown to yield significant improvements in skeletal muscle strength (Meyer et al., 2003; Steiner et al., 2004; Zoll et al., 2006; Gremeaux et al., 2010).

In the COPD population, both ventilatory constraints and locomotor muscle weakness limit patients with severe disease from exercising at intensities high enough to impact muscle strength and performance. As such, EET is an appealing modality because it offers the possibility of overloading the muscle to levels necessary to induce adaptation without also overloading the respiratory system (Rooyackers et al., 1997; Rocha Vieira et al., 2011). Indeed, we have previously shown that the ventilatory requirements to eccentric cycling in patients with severe COPD were similar to conventional CET despite the former exercise being performed at an average 5-times higher mechanical power (Rocha Vieira et al., 2011). Although the feasibility of this modality for severe COPD patients has been established (Rooyackers et al., 2003; Rocha Vieira et al., 2011), the impact of EET on muscle strength and cellular adaptation has not been investigated. Furthermore, whereas previous reports in healthy elderly subjects and patients with coronary artery disease suggest that EET induces signaling pathways involved in promoting muscle hypertrophy but not oxidative capacity (Zoll et al., 2006; Mueller et al., 2011), these mitochondrial measures have not been examined in COPD patients to date. Distinguishing these adaptations is important in order to identify and individualize the most effective rehabilitation strategy for patients with differing COPD disease severities and functional capacities.

Thus, the aim of this study was to characterize the locomotor muscle adaptations to EET in patients with severe COPD, and compare them with adaptations gained through conventional concentric cycle training. We hypothesize that patients undergoing EET will demonstrate larger gains in muscle strength and mass than the CET group when carried out at the same relative heart rate intensity, and that these gains are achieved in part through increases in fiber hypertrophy with no effect on mitochondrial adaptation. To address this aim, we analyzed functional and structural adaptations within locomotor muscle of moderate to very severe COPD patients who were randomized to a 10-week intervention of either EET or CET training.

## Methods

### Participants and study design

Ambulatory men aged 50–80 years with confirmed COPD were recruited from the clinic at the Montreal Chest Institute to participate in a randomized clinical trial (ClinicalTrials.gov identifier: NCT01077102 RCT) comparing eccentric to concentric cycle ergometer training. Patients were diagnosed according to the Global Initiative for Chronic Obstructive Lung Disease (GOLD) based on spirometry values corresponding to moderate (GOLD stage II), severe (GOLD stage III), and very severe (GOLD stage IV) COPD (Global Initiative for Chronic Obstructive Lung Disease, 2017). GOLD stages II through IV are characterized by worsening airflow limitation and in general, worsening shortness of breath, exercise capacity, and muscle function although muscle abnormalities are detectable in patients with mild to moderate COPD (van den Borst et al., 2013). Of the total number of participants enrolled in the RCT (*n* = 24), 15 patients who were willing to undergo a muscle needle biopsy before and after the training intervention are presented here. The ethics review board of the Research Institute of the McGill University Health Centre approved the study. Exclusion criteria included (1) severe or unstable cardiac disease or orthopedic problems that could preclude exercise participation, (2) supplemental oxygen therapy, (3) current or recent (within 3 months) exacerbation (defined as worsening of at least two respiratory symptoms such as dyspnea, sputum production, sputum color) with duration of three or more days of systemic steroid or antibiotic use), (4) participation within a pulmonary rehabilitation program within the preceding year. Eligible participants provided informed consent and underwent an initial medical screening, and then were randomly assigned to the CET or EET group. Prior to any training, all patients underwent pulmonary function testing, cardiopulmonary exercise testing, body composition assessment, quantitative muscle dynamometry, and a muscle needle biopsy at least 1 week prior to the commencement of training at the Montreal Chest Institute and the Department of Kinesiology, McGill University. These procedures were repeated after 10 weeks of training.

### Exercise training

The COPD patients were randomized into an eccentric cycling (EET) or a concentric cycling (CET) group, both whom performed the exercise on the same custom-built recumbent cycle ergometer (Strasbourg, France) in the exercise physiology lab of the Montreal Chest Institute. For the EET, the pedals were driven in a backward direction by an electric motor, and the patient had to maintain a pedaling frequency of 60 rev-min^−1^ by exerting a given force against the pedals. Direct visual feedback related to pedaling frequency and the measured mechanical power was provided through computer software. For the CET, patients pedaled in a forward direction at a similar frequency with feedback.

The training protocol was set at three sessions per week of 30 min at a target intensity that was derived from our previous feasibility trial (Rocha Vieira et al., 2011): for the concentric group, the target cycling power was 60–80% peak workrate achieved during the baseline incremental cardiopulmonary exercise test; for the eccentric group, target intensity was equivalent to four times the power output corresponding to 60–80% peak workrate of the baseline incremental cardiopulmonary exercise test (in the concentric mode). According to our feasibility study, this estimation allowed for both the EET and CET groups to exercise at a similar relative HR intensity. In order to prevent muscle damage and soreness particularly in the EET, all patients underwent a familiarization period for the first 2 weeks where the intensity was 20–40% of their target for either 20 min (week 1) or 30 min (week 2). Therefore, the exercise duration was maintained at 30 min while the intensity was progressively increased to the target level. Each patient did a 5-min warm-up of unloaded pedaling prior to starting each session. Heart rate and ratings of perceived exertion (RPE) using the Borg CR10 Scale for dyspnea and leg pain were monitored throughout the exercise by the supervising kinesiologist and research assistant. Training compliance was set at greater than or equal to attending 80% of the sessions during the training period.

### Outcome measures and experimental procedures

#### Peak aerobic power

Participants performed a standard symptom-limited peak incremental exercise test according to established guidelines (Ross, 2003) on the recumbent cycle ergometer (Strasbourg, France) in the concentric mode. Power was increased by 5–10 watts/min until exhaustion and peak aerobic power was recorded for the calculation of cycle training intensity. Heart rate, blood pressure, and oxyhemoglobin were monitored throughout the test, and peak oxygen consumption (VO_2_) and ventilation were assessed at baseline (MediSoft, Sorinnes, Belgium).

#### Body composition

Dual-energy x-ray absorptiometry (DEXA) was performed on the GE Lunar iDXA scanner (General Electric Healthcare, USA) to measure the total and regional body composition (fat and lean mass content) before and after training. Briefly, thigh-specific lean mass and fat content were determined using regions of interest (ROI) with GE Lunar Encore™ software (V.11.20) by subtracting the lower leg ROI below the tibio-femoral joint from the total leg mass (distal section of the leg below the iliac crest and femoral neck).

#### Serum creatine kinase levels

Blood was obtained using standard arm venipuncture for analysis of serum creatine kinase, a marker of muscle damage at baseline, after 2, 5, and 10 weeks of training in both groups.

#### Muscle strength

Assessment of the quadriceps muscle isometric peak torque was done before and after training using a quantitative Biodex System 4 Dynamometer (Biodex Medical Systems Inc., New York). Following familiarization with the device and testing procedures, patients were instructed to produce a maximal effort contraction of 4 s with the knees flexed at 60°. After a 10 min rest, patients were asked to perform five sequential volitional maximal contractions over a range of motion from 15° to 100° at 60°/s to calculate the total isokinetic work, which was taken as the sum of the five contractions. Each testing procedure was performed twice with the right leg and the highest value was used in the analysis.

#### Muscle biopsy outcomes

A biopsy of the left *vastus lateralis* at the mid-thigh level was taken before training and within 4–7 days after the last training bout using the modified Bergstrom needle technique with suction (Shanely et al., 2014). An ~200 mg sample of muscle was obtained and portioned out for the various analyses described below. With the exception of the mitochondrial respiration assay which required fresh tissue, muscle was immediately snap frozen in liquid nitrogen or frozen using liquid Isopentane (cooled in liquid nitrogen) for *in-situ* labeling, protein western blot analysis, and mitochondrial enzymatic activity. These samples were stored at −80°C until analysis.

#### Analysis of mitochondrial biogenesis transcript

To detect activation of mitochondrial biogenesis, we quantified levels of peroxisome proliferator-activated receptor γ coactivator 1α (PGC-1α) mRNA in muscle samples as recently described (Konokhova et al., 2016). Total RNA was extracted from samples using the RNeasy Lipid Tissue Mini Kit (Qiagen), according to manufacturer' s instructions. RNA concentration and purity (A 260/A 280 ratios >1.8) were assessed using a spectrophotometer. RNA (1 μg) was reverse transcribed to cDNA using qScript™ cDNA Synthesis Kit (Quanta BioSciences) according to the manufacturer' s instructions. The PPARGC1alpha (PGC-1α) primer was designed with Primer 3 plus [forward TTTCCTTTTGCCATGGAATC, reverse: GAAAGAACCGCTGAACAAGC (NCBI ref: XM_005248130.1)]. TATA box binding protein [TBP; primers: forward TATAATCCCAAGCGGT TTGC-3, reverse GCTGGAAAACCCAACTTCTG (NCBI ref: NM_001172085.1)] was used as the reference gene for quantification of mRNA levels. The cDNA was amplified using Power SYBR® Green PCR Master Mix (Life Technologies) as previously described (Konokhova et al., 2016). All real-time PCR experiments were performed in triplicates along with melt curve analysis to assess primer dimer formation or contamination. Then, the comparative CT method was used to calculate fold changes in expression in the COPD patients post-training and compared to baseline levels, where ΔCT = CT of PGC-1α gene − CT of TBP and ΔΔCT = ΔCT of patients prior to training − ΔCT of patients following the training intervention.

#### *In-situ* labeling

Muscle sections were cut at either 8 or 10 μm thickness using a cryostat (−20°C) and mounted on a slide and air-dried. Pre and post training analyses for each patient were done on similar thickness sections.

To evaluate evidence of muscle damage, a standard hematoxylin and eosin stain was performed. To detect fiber type and analyze fiber cross sectional area, immunofluorescent labeling for myosin heavy chain slow isoform (MHC type 1) expression and myosin heavy chain fast isoform (MHC type 2a and 2x) expression was performed as we have previously described (Konokhova et al., 2016). Muscle sections were blocked in 10% normal goat serum (Life technologies, 50-062Z) and incubated with primary antibodies (from Developmental Studies Hybridoma Bank) for 1 h at RT at specified working dilutions: BA-F8 (mouse anti-myosin heavy chain (MHC)1 IgG2b, 1:25), Sc-71 (mouse anti-MHC2a IgG1, 1:200), and 6H1 (mouse anti-MHC2x IgM, 1:25). Following three washes in PBS, sections were probed with the following secondary antibodies: Alexa Fluor 350 IgG2b (y2b) goat anti-mouse 1:500, Alexa Fluor 594 IgG1 (y1) goat anti-mouse 1:100, Alexa Fluor 488 IgM goat anti-mouse 1:500, and Alexa Fluor 488 IgG goat anti-rabbit 1:500. Sections were then washed in PCS and mounted using ProLong gold. Images were captured on a Zeiss Axio Imager M2 fluorescent microscope (Zeiss, Germany). To detect evidence of mitochondrial biogenesis *in situ*, we probed for PGC-1α content (Supplementary Figure 1). Muscle sections were thawed and fixed in pre-cooled Acetone for 15 min at 4°C, washed in TBS (3 × 5 min). Muscle tissue was then permeabilized with 0.1% Triton (Life Technologies; 28314) for 15 min at room temperature, washed with TBS (3 × 5 min) blocked in 10% normal goat serum for 15 min at room temperature. Sections were then probed for PGC1α (AB3242, Millipore, 1:50 dilution) overnight at 4°C. Following primary incubation, muscle sections were washed in TBS (3 × 5 min) and exposed to Alexa Fluor 488 IgG at 1:500 dilution (goat anti rabbit) for 90 min at room temperature, while protected from light. Sections were washed again (3 × 5 min TBS) and mounted with Vectashield mountant (Vector Laboratories, H-1200, Burlingame, CA, USA). Given the fiber-type variability of PGC-1α content, for this determination we used the muscle cross-section that was cut immediately following those used for MHC immunolabelling as we have previously described (Gouspillou et al., 2014b).

#### Imaging and image analysis

All images were collected using an upright Zeiss microscope (Carl Zeiss, Germany). Tiled images of the entire muscle biopsy cross-section were obtained at 10x magnification. The entire cross-section was imaged and then analyzed in Fiji software, an open source NIH platform for biological image analysis (Schindelin et al., 2012) to determine individual fiber cross-sectional area (CSA, μm^2^) and to identify pure MHC slow (type 1) myofibers, MHC fast (type 2a) and MHC fast (type 2x) as well as MHC co-expressing myofibers (more than one MHC signal). Myofibers that were negative for the other MHC types were categorized as expressing just the specific pure MHC, whereas those myofibers having a positive signal in more than one channel were categorized as co-expressing. On average, 455 ± 226 fibers per patient were counted pre-training and 331 ± 180 fibers per patient were counted post-training. For the other labeling, 100 or 200x magnification was used. PGC-1α was quantified using densitometry analysis as previously described (Gouspillou et al., 2014b). Briefly, individual fibers were traced using ImageJ software (NIH, USA) and once all the fibers were traced, the mean gray intensity for each fiber was determined. Fibers were then matched for MHC signal in a serial muscle cross-section to obtain fiber type specific data. To detect if training resulted in muscle damage, H&E labeling was analyzed for mononuclear infiltration, streaming, and necrosis by a trained specialist blinded for patient group and biopsy time in the Neuromuscular Disease Department of the Montreal Neurological Institute.

#### Electron transport complex content

Western blot analysis was used to measure protein content of the mitochondrial oxidative phosphorylation system located on the inner mitochondrial membrane and the Voltage-dependent anion channel (VDAC) located on the outer mitochondrial membrane. Approximately 30–40 mg of muscle was homogenized in 10 vol of an extraction buffer composed of 50 mM Tris base, 150 mM NaCl, 1% Triton X-100, 0.5% sodium deoxycolate, 0.1% SDS, and 10 μl/ml of a protease inhibitor cocktail (P8340, Sigma). The homogenate was centrifuged at 15,000 g for 15 min at 4°C. Protein content in the supernatant was determined using the Bradford method with BSA as standard. Aliquots of supernatant were mixed with Laemli buffer and subsequently boiled at 95°C for 5 min. Sixteen micrograms were loaded into 12% gels, electrophoresed by SDS-PAGE, and then transferred to polyvinylidene fluoride membranes (Life Sciences). Membranes were incubated for 1 h at room temperature in a blocking buffer composed of 5% (w/v) non-fat dried milk in Tris-buffered saline containing 0.1% Tween 20 (TBS-T) and probed overnight at 4°C with primary antibodies for mouse monoclonal anti-VADC (Ab14734, 1:1500) and mouse polyclonal OXPHOS (Ab110413, 1:2000) diluted in 5% non-fat milk. The following day, membranes were washed in TBS-T and incubated with appropriated Horseradish Peroxidase (HRP)-conjugated secondary antibodies diluted in 5% BSA for 1 h at room temperature. Protein signals were detected using enhanced chemiluminescence substrate (24080; Thermo Scientific; Waltham, MA, USA), imaged with a G-Box chem imaging system and analyzed by GeneSys software (Syngene, Cambridge, UK). Ponceau staining was used to ensure equal protein loading. VDAC and OXPHOS protein content was normalized for Ponceau staining that was imaged and analyzed with the same system.

#### Mitochondrial respiration assay

Permeabilized myofiber respiration was assessed with a polarographic oxygen sensor (Oxygraph-2k; Oroboros, Innsbruck, Austria) calibrated as required for O_2_ concentration, environmental variables and auto-O_2_ consumption as routinely performed in our laboratory (Picard et al., 2010; Gouspillou et al., 2014a). Briefly, to prepare muscle bundles, fibers were manually teased apart and permeabilized using buffer A mixed with 0.05 mg/ml saponin and then placed in buffer B for respiration. These experiments were performed at 37°C and analyzed with a specially developed program using Igor Pro software (Wavemetrics). Baseline respiration was measured by placing 3–6 mg (wet weight) permeabilized bundles into the respirometer. Thereafter, the substrate protocol assessing O_2_ flux was added sequentially as follows, with each step interspersed with a period of stabilization between injections: 10 mM glutamate +5 mM malate +2 mM ADP (maximal ADP-stimulated respiration, State 3, with complex I substrates), +10 mM succinate (Succ; State 3, with complex I and II substrates), 10 μM antimycin A (AA), and 5 mM ascorbate +0.5 mM N,N,N′,N′-tetramethyl-p-phenylenediamine (TMPD). Mitochondrial respiration was analyzed in a subset of patients (*n* = 4 per group) due to insufficient tissue extraction from all biopsies.

### Statistics

The sample size was calculated based on a similar study assessing changes in muscle strength after 8 weeks of EET and CET in patients with coronary artery disease (Steiner et al., 2004). Using this and taking into account a 15% patient drop out rate, we predicted that with 7 patients in each group (*n* = 14), we could show a difference in muscle strength between groups. Statistical analysis was performed using GraphPad Prism (Prism 7, GraphPad Software, Inc.). Results are presented as mean ± SEM, with significance set at *P* < 0.05. Data was tested for normality using the Pearson omnibus normality test. Two-tailed paired Student's *t*-tests were applied to analyze changes from pre- to post-training within a group, whereas unpaired Student's *t*-test was used to detect intergroup differences. A repeated measures analysis of variance (ANOVA) was used to determine training effect on PGC-1α content within the different fiber types.

## Results

### Baseline characteristics

Recruitment and randomization from the larger RCT yielded eight COPD patients in the EET group and seven patients in the CET group (Table 1). A greater number of patients in the EET group (87.5%) had more severe airflow obstruction (GOLD III and IV) compared to CET group (57%). All patients were on bronchodilators, and there were no differences in use of other medications between groups (See Supplementary Table 1).

**Table 1:** **Demographic and baseline characteristics of COPD patients by training group**.

**Characteristic**	**EET (*n* = 8)**	**CET (*n* = 7)**
Age (years)	68 ± 2	63 ± 2
Height (cm)	170 ± 3	173 ± 2
Weight (kg)	76 ± 6	79 ± 8
BMI (kg/m^2^)	26.5 ± 1.9	26.7 ± 2.8
FFMI (kg/m^2^)	17.5 ± 0.5	18.5 ± 1.0
FEV_1_ (% Pred. Pβ_2_A)	36.2 ± 3.7	45.8 ± 5.0
FEV_1_/FVC (% Pred. Pβ_2_A)	34.4 ± 3.7	37.2 ± 1.7
GOLD Stage II	1	3
GOLD Stage III	4	3
GOLD Stage IV	3	1
Steps per day	3372 ± 869	4271 ± 655

*Data are presented as means ± SE except disease categories where the values are frequency counts. BMI, Body Mass Index; FFMI, Fat-free mass Index (kg/m^2^); FEV_1_, Forced expiratory volume in 1 s; FEV_1_/FVC, Forced expiratory volume in 1 s over forced vital capacity; GOLD, Global Initiative for Chronic Obstructive Lung Disease, a classification of airflow limitation severity in COPD based on post-bronchodilator FEV_1_; Stage II (moderate COPD) characterized by FEV_1_/FVC <70%; 50% <FEV_1_ <80% predicted; Stage III (severe COPD) characterized by FEV_1_/FVC <70%; 30% <FEV_1_ <50% predicted; Stage IV (very severe COPD) characterized by FEV_1_/FVC <70%; FEV_1_ <30% predicted. Pβ_2_A, Post β_2_Agonist*.

### Exercise training compliance, progression, and safety

All 15 patients who agreed to undergo a baseline muscle biopsy completed the 10-week training program and compliance was similar between groups (EET = 87.0 ± 6%; CET = 85.7 ± 6% of all sessions, *P* = 0.66). During the training, one patient in the EET group experienced an acute exacerbation and one patient in the CET group experienced low back pain that resulted in their training programs being delayed by 1 week and 9 days, respectively, until their health status improved. Two patients in the EET group experienced knee pain during training but were able to complete training. One patient in the CET group experienced hip pain but was also able to complete the study.

**Figure 1:**
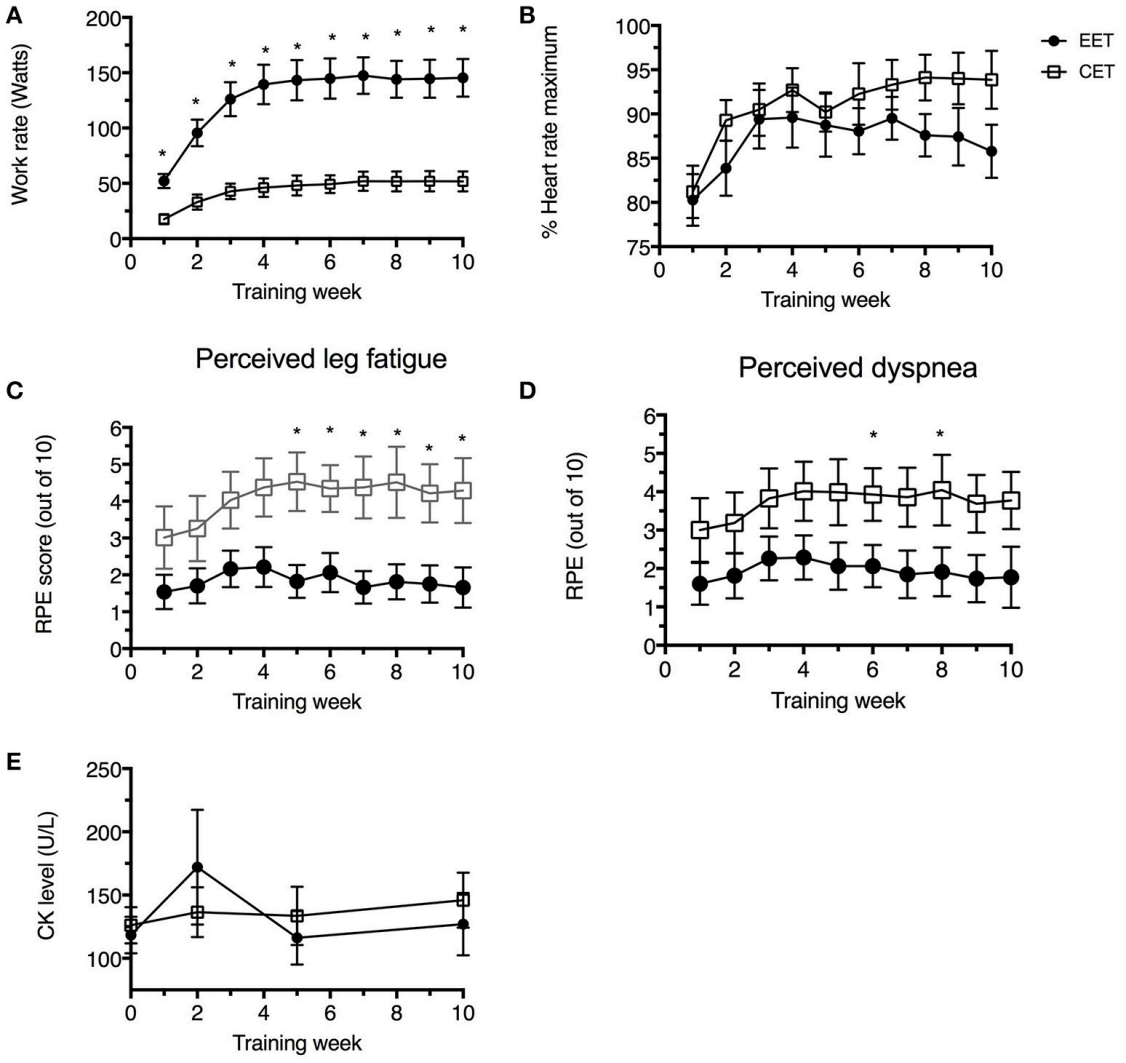
**Data obtained over 10 weeks of training in the EET (closed circles) and CET (open squares) groups. (A)** Mean power output expressed in Watts per week; **(B)** heart rate expressed as a percentage of baseline maximal heart rate obtained at the end of the incremental cardiopulmonary exercise test; **(C)** Ratings of perceived exertion (RPE) using the Borg CR10 scale for leg fatigue during each training session; **(D)** Ratings of perceived exertion using the Borg CR10 scale for dyspnea during each training session; **(E)** serum CK levels at four timepoints. Weekly values for **(A–D)** represent the average of three exercise sessions per week per patient. Values are means ± SEM. ^*^Significantly different compared to other group at the same time point, *p* < 0.05.

As shown in Figure 1A, the training load was significantly higher in the EET group compared to the CET group during week 1, and this load was progressively increased over the first 4 weeks in both groups until it reached a plateau. By the last week of training (week 10), the mean training load of the EET group was three-times higher (145 ± 17 watts) than the CET group (52 ± 9 watts, *P* < 0.01). Despite the different training loads, the relative heart rate intensity (% peak attained at the pre-training assessment) was similar between groups throughout the 10-week training program (between 80 and 95% peak HR, Figure 1B). Significant statistical and clinical differences were demonstrated for perceptions of leg fatigue (Figure 1C) and dyspnea (Figure 1D) where patients in the EET group reported a lower perception of leg fatigue and dyspnea than the CET group throughout the training program.

For both groups, the level of serum CK was normal at baseline and fluctuated within the laboratory normal range (5–140 U/L) during the training period (Figure 1E).

### Body composition

There were no changes in body weight or BMI in either group after the training period. However, as detected by DEXA, the EET group experienced a significant decrease in body fat percentage (pre 32.0 ± 3.9%; post 30.7 ± 3.6%, *P* < 0.05) and a trend toward increase in total body lean mass content (pre 47.5 ± 2.0 Kg; post 48.3 ± 1.9 Kg, *P* = 0.07), whereas no such reductions in body fat (pre 28.3 ± 4.7%; post 27.7 ± 4.4%, *P* = 0.14) or increases in lean mass (pre 52.8 ± 2.8%; post 52.2 ± 2.9%, *P* = 0.13) were seen in the CET group. Additionally, similar findings were detected with respect to the thigh: the EET group experienced a decrease in thigh fat percentage (pre 30.0 ± 2.5; post 27.9 ± 2.1 Kg, *P* < 0.01) and increase in relative thigh muscle mass (pre 69.3 ± 2.6%; post 71.3 ± 2.3%, *P* < 0.01), with no improvements detected in the CET group (thigh fat percentage pre 24.5 ± 3.3; post 24.5 ± 3.1%, *P* = 0.92) or thigh muscle mass (pre 75.1 ± 3.8%; post 75.1 ± 3.6%, *P* = 0.93).

### Quadriceps muscle strength

**Figure 2:**
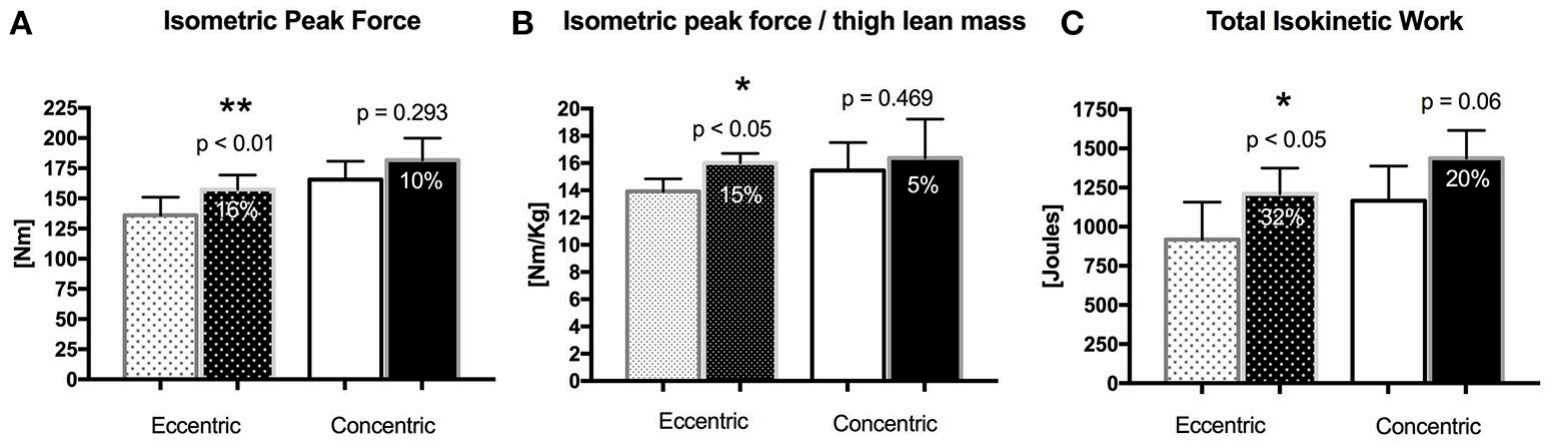
**Changes in quadriceps muscle strength in the EET and CET after training (dark bars). (A)** Isometric peak strength; **(B)** Isometric peak strength normalized for thigh lean mass; **(C)** Total isokinetic work. Bar values are means ± SEM ^**^Significant difference compared with pre-training values, *p* < 0.01. ^*^Significant difference compared with pre-training values, *p* < 0.05.

EET led to significant increases in isometric peak strength as well as in isometric strength normalized to thigh lean mass (Figures 2A,B), whereas no such increases were detected in the CET group. Total isokinetic work (Figure 2C) also increased significantly post training in the EET group, while there was a trend for increase in the CET group.

### Cardio-pulmonary exercise testing

Peak workrate measured during standard incremental cardiopulmonary exercise testing did not increase significantly in the EET group (pre 49.0 ± 6.7; post 54.0 ± 5.8 watts, *P* = 0.133) but did in the CET group (pre 62 ± 10; post 73 ± 10 watts, *P* < 0.05). However, both groups achieved the minimum clinically important difference for peak workrate (5 watts) during incremental exercise testing as defined by Sutherland and Make (Sutherland and Make, 2005). Technical failure of the metabolic cart prevented measurement of peak VO_2_ after training, and thus, assessment of aerobic training response, in both groups.

### Muscle ultrastructure and central nuclei

**Figure 3:**
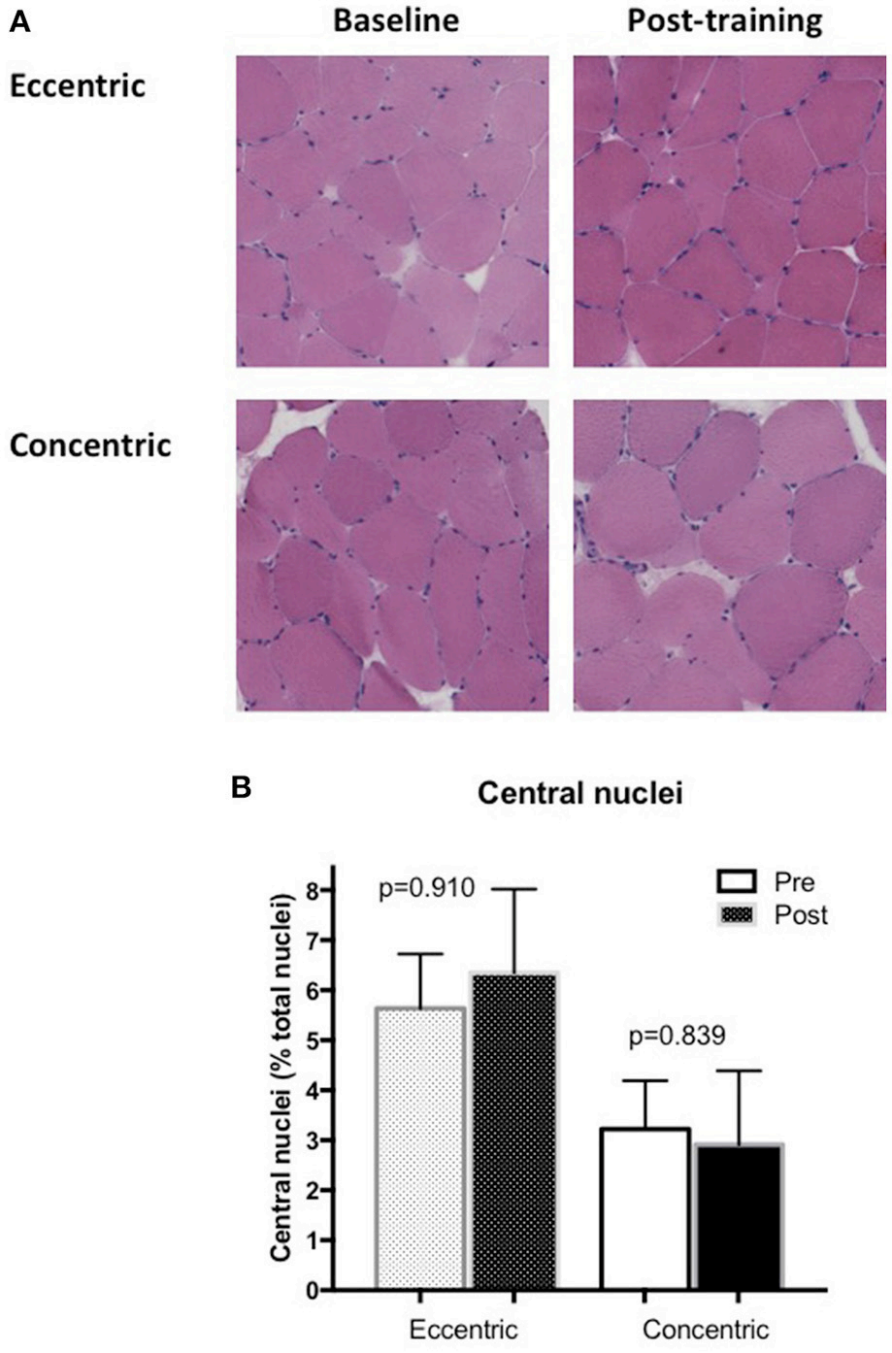
**(A)** Representative muscle biopsy images stained for hematoxylin and eosin at baseline and after training in the EET and CET group. **(B)** The proportion of central nuclei relative to total nuclei in vastus lateralis sections labeled with DAPI and Laminin pre and post biopsy for the EET and CET groups.

Qualitatively, normal muscle ultrastructure was observed in biopsies of both the EET and CET groups (Figure 3A), and there was no evidence of increased inflammation or necrosis in the post-training samples. The proportion of central nuclei relative to total nuclei (reflecting muscle degeneration/regeneration) did not increase after training (Figure 3B) in either the EET (pre 5.6 ± 1.1%; post 6.4 ± 1.7%, *P* = 0.910) or CET (3.2 ± 1.0; post 2.9 ± 1.5%, *P* = 0.839) groups and did not differ between the groups in the post training biopsy (*P* = 0.279). However, there was one statistical outlier detected in the EET group post training, with 17% of the nuclei being centralized vs. in the periphery. Data for this participant was kept in for the analysis as it did not alter the significance of the conclusions.

### Muscle fiber size and type

**Figure 4:**
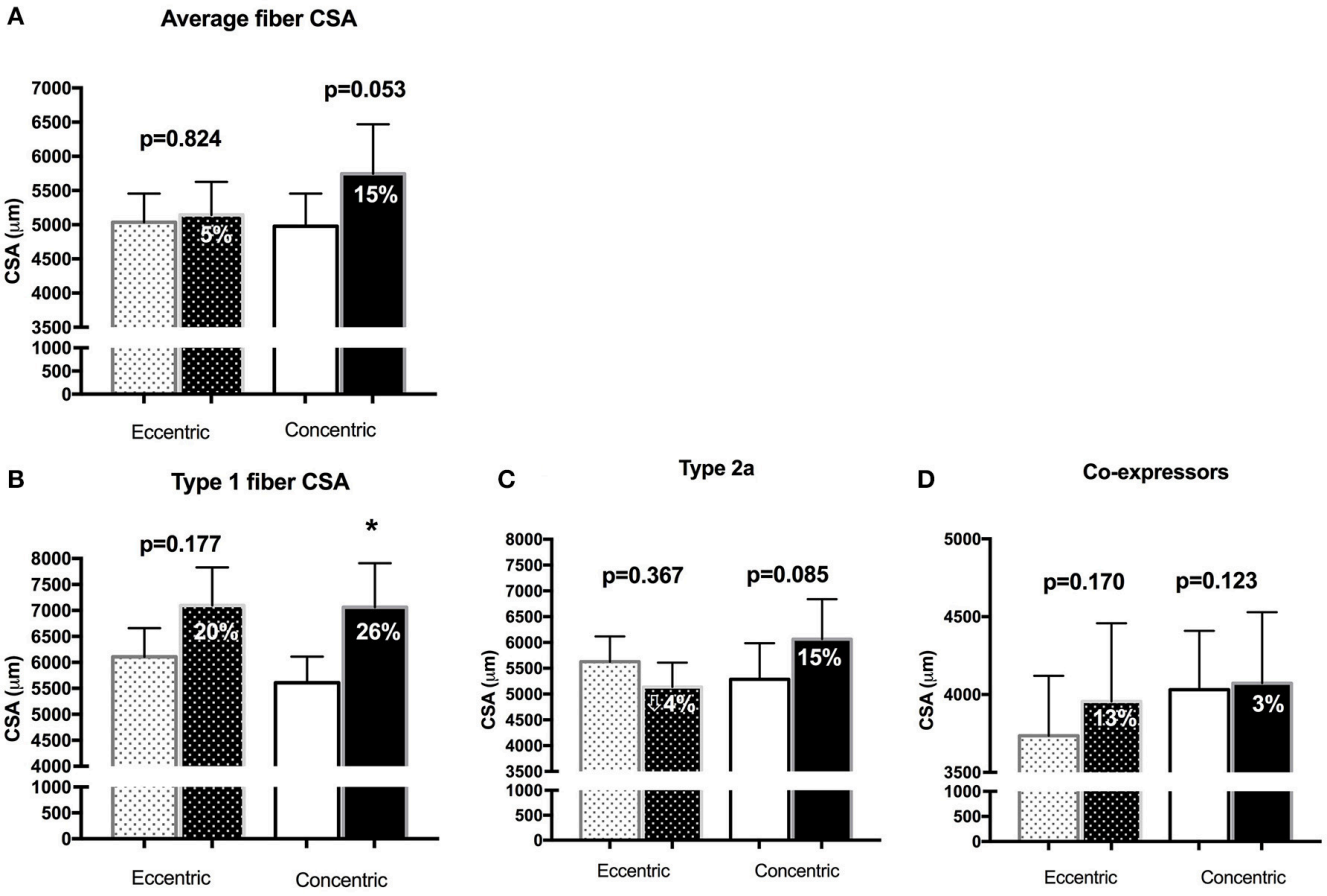
**Morphometrical analysis of fiber cross-sectional areas (μm^**2**^) for (A)** the average fiber (not taking fiber type into account); **(B)** pure Type 1 fibers; **(C)** pure Type 2a fibers; **(D)** fibers that express more than one myosin heavy chain isoform in the Eccentric and Concentric group. The darker shaded bars represent the post-training value, with the percentage reflecting the mean of the percent change per subject Bar values are means ± SEM. ^*^p < 0.05.

Muscle morphological characteristics are reported in Figure 4. The average fiber CSA did not change after the intervention in the EET group (pre 5035 ± 420 μm^2^, post 5141 ± 484 μm^2^, *p* = 0.824), with variability in both the magnitude and direction of the fiber-type specific responses (Type 1 CSA from 6109 ± 551 to 7095 ± 735 μm^2^, *p* = 0.177, mean ± SEM of difference per subject = 20 ± 14%; Type 2a CSA from 5628 ± 491 to 5137 ± 471, *p* = 0.367, mean ± SEM of difference per subject = −4 ± 10%; co-expressing fiber CSA from 3735 ± 1091 to 3955 ± 1421, *p* = 0.170, mean ± SEM of difference per subject = 13 ± 15%). Of note, the one patient in the EET group with the outlier value of centralized nuclei was close to being a statistical outlier for average fiber CSA, demonstrating a 43% decrease after eccentric training whereas the overall group change was 5% (25% Standard deviation). In the CET group, increases were detected in mean CSA (4977 ± 479 to 5744 ± 725, *p* = 0.053, mean ± SEM of difference per subject = 15 ± 5%), Type 1 fiber (5607 ± to 7062 ± 847, *p* = 0.036, mean ± SEM of difference per subject = 26 ± 9%), Type 2a (5286 ± 700 to 6065 ± 774, *p* = 0.085, mean ± SEM of difference per subject = 15 ± 9%) with little change in coexpressing fibers (4031 ± 1000 to 4072 ± 1208, *p* = 0.123, mean ± SEM of difference per subject = 3 ± 10%). Fiber type proportions (Type 1, Type 2a, Type 2x) were not significantly altered with either modality of training (Supplementary Table 2).

### Muscle mitochondrial adaptations

**Figure 5:**
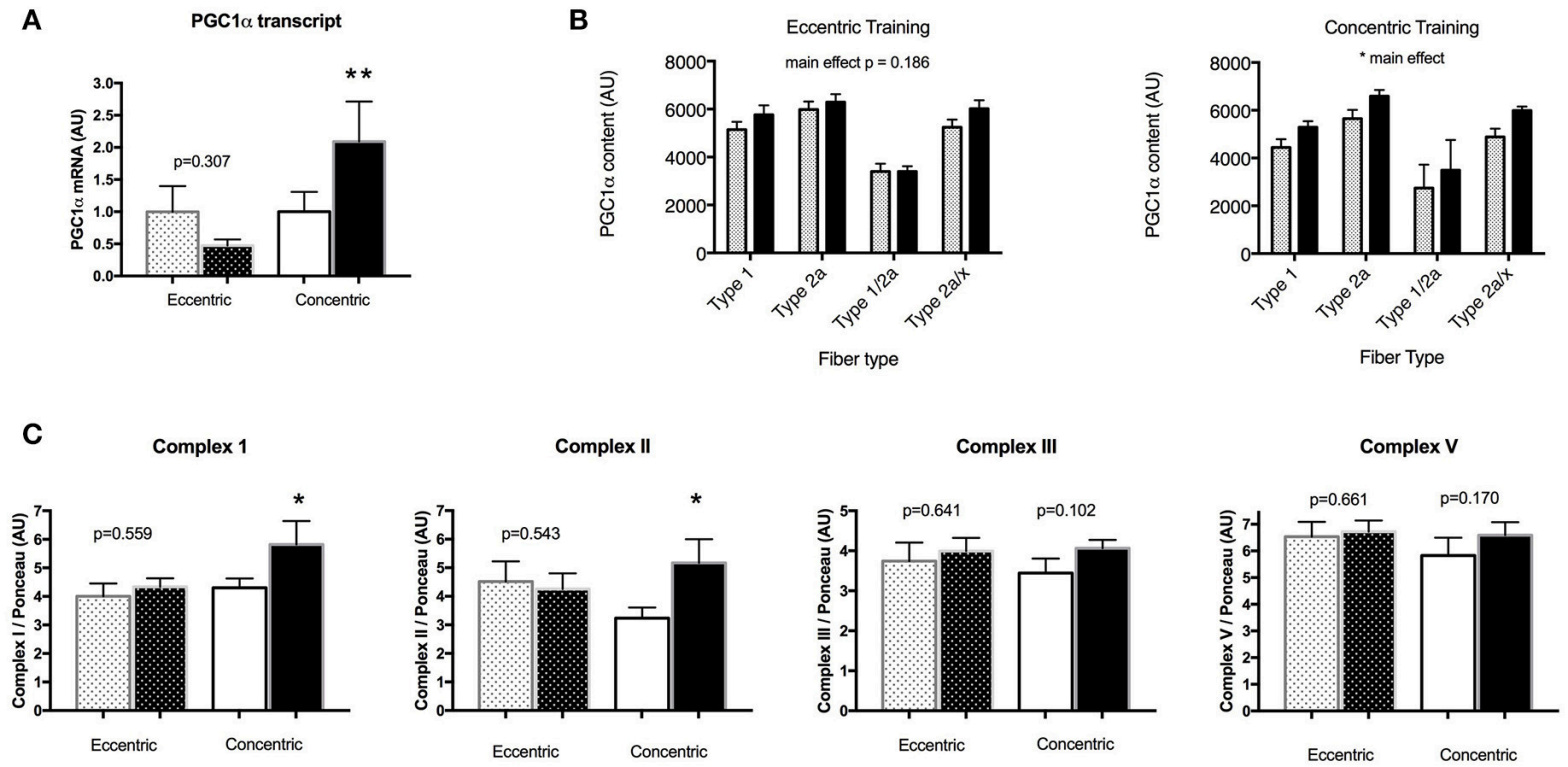
**Changes in markers of mitochondrial biogenesis and content before (open bars) and after (dark bars) training in COPD patients. (A)** EET had no effect on PGC-1α mRNA in contrast to CET. Similarly, **(B)** EET had no effect on PGC-1α content as detected *in situ* within specific fiber types, whereas CET led to an overall increase in PGC-1α content. **(C)** Analysis of electron transport chain complexes revealed no effect of EET, whereas concentric training increased both Complex I and Complex II content. Bar values are means ± SEM. ^**^Significant difference compared with pre-training values, *p* < 0.01.^*^Significant difference compared with pre-training values, *p* < 0.05.

There was no change in transcript levels of the key mitochondrial biogenesis regulatory factor PGC-1α after EET (Figure 5A). In contrast, in the CET group post-training sample, PGC-1α mRNA was two-fold that of the pre-training value (*P* < 0.05). Consistent with this finding, PGC-1α protein levels reflected by densitometric analysis of immunolabeled muscle fibers did not change in the EET group within any fiber type, however there was a main effect of training in the CET group, with higher PGC-1α content in Type 1, Type 2a, and co-expressing fibers after training (*P* < 0.05, Figure 5B).

**Figure 6:**
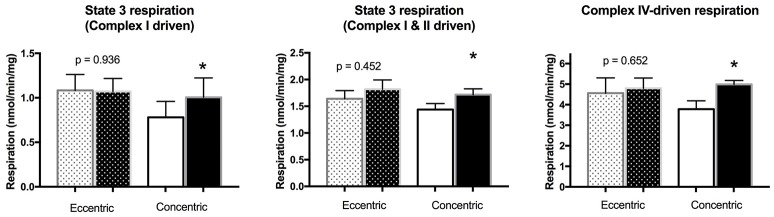
**Changes in mitochondrial respiration in permeabilized myofibers of a subset of patients in both the EET (***n*** = 4) and CET (***n*** = 4) groups**. Eccentric ergometer training had no effect, while concentric cycle training increased State 3 (Complex 1 driven), State 3 (Complex I and II driven), and Complex IV-driven respiration within locomotor muscles of COPD patients. ^*^Significant difference compared with pre-training values, *p* < 0.05.

Analysis of electron transport chain complexes revealed no changes in the EET group, but significant increases in levels of Complex 1 and Complex II protein in the CET group (Figure 5C). We had sufficient tissue to measure mitochondrial respiration in saponin-permeabilized fibers in 4 patients per group, revealing no effect of EET on the various states of respiration (Figure 6). However, significant increases after CET were detected for maximal ADP stimulated respiration with both complex I (state 3 respiration GM) and complex I and II (state 3 GMS), and an increase in maximal respiration through Complex IV (V'ascorbate + TMPD).

## Discussion

This study is the first to detail the functional and cellular responses of locomotor muscle to 10 weeks of eccentric cycle training in patients with moderate to severe COPD, and contrast them to conventional endurance training. Patients were able to overload their muscles, on average, at a workload three times that of CET while maintaining a similar relative heart rate and without experiencing undue leg fatigue or breathlessness. Consistent with our hypothesis, EET led to a significant increase in peak muscle strength whereas CET did not; however, this functional gain was not associated with a significant increase in average fiber cross sectional area. Moreover, detailed measurements of mitochondrial biogenesis, content and function in locomotor muscles revealed a lack of adaptation to the eccentric cycling that contrasted with the significant improvements in these parameters with CET. Taken together, these contrasting muscle adaptations to EET vs. CET in severe COPD suggest it would be worthwhile to evaluate a sequential paradigm of eccentric followed by concentric cycling as a means of augmenting the training response and skeletal muscle dysfunction in patients with advanced COPD.

### Eccentric cycling effects on locomotor muscle strength and hypertrophy in COPD

The use of dynamic eccentric exercise is increasingly being proposed as a therapeutic strategy for individuals with reduced tolerance for physical activity, due to the ability of eccentric muscle actions to produce a higher power output for a given metabolic, neural, and cardiorespiratory cost compared to concentric muscle actions (Hortobagyi and DeVita, 2000; Roig et al., 2008). More specifically, eccentric cycle exercise training paradigms (whereby the eccentric load is applied continuously over the pedaling action for time periods consistent with aerobic exercise) have been shown to result in greater gains in muscle strength than paradigms consisting predominantly of concentric muscle actions in healthy young (LaStayo et al., 2000, 2003), elderly (Mueller et al., 2009), in patients with coronary artery disease (Steiner et al., 2004) and other clinical conditions (Isner-Horobeti et al., 2013). In the COPD population, only one previous study has assessed the impact of eccentric cycle training, using parameters (10 weeks of cycling at 160 watts) similar to those in our study but in conjunction with a mixed exercise training program consisting of aerobic interval training and strengthening exercises (Rooyackers et al., 2003). The authors reported improvements in cardiovascular fitness and pulmonary diffusing capacity in the patients who underwent both EET and general exercise training but not in the patients who only did the latter. Importantly, no measurements of muscle function were included in this prior study. In our current study, we report significant increases in measures of peak muscle strength in COPD patients after EET alone. Given the prevalence of muscle weakness estimated at 30–40% (Seymour et al., 2010) in this patient population (which tends to affect individuals of middle to advanced age) and its compromising effects on physical performance and functional ability, along with the notion that muscle strength is important in maintaining functional autonomy in the elderly (Frontera et al., 1988), our findings support the use of EET as a therapeutic strategy for countering muscle weakness in COPD.

We expected muscle hypertrophy to accompany the increase in peak strength after EET, a hypothesis based on previous findings in healthy muscle showing that when eccentric and concentric actions are pre-formed at the same metabolic level during cycle ergometer exercise, the former resulted in a greater average muscle fiber CSA (LaStayo et al., 2000). In our COPD patients however, average fiber CSA following EET did not change from baseline levels and was characterized by both a large variability in the directionality of fiber-type specific responses to eccentric training as well as high inter-subject variability in CSA response. Although not statistically significant, increases in type 1 fiber (20 ± 14%) and co-expressing fiber (13 ± 15%) CSA were detected, whereas type 2a CSA decreased (−4 ± 10%). An increase in type 1 fiber CSA would theoretically be expected given the dynamic modality of the exercise whereby the less fatigable fibers would be recruited throughout the exercise session, whereas the divergent responses of type 2a and co-expressing fibers may suggest a transitioning and remodeling of those fiber types muscle due to the training. The 20% increase in type 1 fiber CSA may be biologically meaningful, particularly given the inter-subject variability in response. Of note is one patient in the EET group who demonstrated a large decrease (−45%) in CSA, which corresponded to the post-training biopsy findings indicating the highest level of central nuclei (17%). This patient was classified with very severe COPD (Stage 4) and had one of the lowest FFMI and cardiorespiratory fitness levels at baseline. Whether the variability in response is reflective of the exercise stimulus or the greater number of patients with more severe COPD in the EET group is not clear. Despite randomization, there was an unequal distribution of GOLD Stage 4 patients, with 3 patients in the EET group compared to only 1 in the CET group. Further to this point, although two of the three patients demonstrated decreases in CSA, the one patient with Stage 4 disease in the CET group exhibited an increase in CSA. Indeed, variability in response to exercise rehabilitation is not uncommon in this patient population (Vogiatzis et al., 2011).

Interestingly, in patients with coronary artery disease, EET resulted in a lack of change in average fiber CSA, despite increases in quadriceps muscle strength (Steiner et al., 2004). These findings led the authors to assume that gains in neural coordinative factors accounted for the strength improvement. These findings are similar to our observations, and although neural factors were not measured in either study, we can only assume that a neural improvement accounted for a portion of the increase in strength after EET in our COPD patient group. However, we cannot discount the notion that physiological adaptations, that are known to occur in response to more classical eccentric resistance training, occurred within the muscle of our patients. Recent studies have reported differential muscle growth patterns in response to eccentric and concentric resistance type training of the quadriceps muscle in humans, whereby eccentric training promotes longitudinal muscle growth due to the addition of new sarcomeres in series, and concentric training promotes transversal hypertrophy with addition of sarcomeres in parallel (Reeves et al., 2009; Baroni et al., 2013; Franchi et al., 2015). This explanation is plausible given the increase in muscle mass observed after EET without an increase in fiber CSA. Differences in the regional distribution of hypertrophy have also been reported, with eccentric training resulting in preferential hypertrophy of the distal region of the vastus lateralis, and concentric training impacting predominantly the midbelly (Seger et al., 1998; Franchi et al., 2014). These findings were based on the use of ultrasonography to determine changes in muscle architecture at multiple locations. Finally, classical eccentric resistance training has been reported to induce extracellular matrix remodeling, increase tendon stiffness, and improve force transmission, all of which may contribute to increasing muscle strength (Heinemeier et al., 2007; Duclay et al., 2009; Mueller et al., 2011). In our current study however, we are not able to discern the predominant mechanism(s) underlying the gains in muscle strength following EET.

In contrast to the EET group, significant increases in CSA were detected in type 1 fibers of the CET group, despite the lower muscle workload over the course of 10 weeks. While concentric exercise training does not typically lead to an increase in fiber CSA in healthy individuals, our finding is consistent with other studies using conventional cycle training in COPD patients that report increases in the average fiber (13%) and type 1 fiber (10–16%) CSA (Vogiatzis et al., 2005, 2010). This is likely attributable to the relative state of muscle disuse in COPD patients given their sedentary behavior. The increase in fiber CSA in CET did not translate into detectable improvements in muscle strength, however peak workrate measured during standard incremental cardiopulmonary exercise did improve, also consistent with previous reports in COPD.

EET had a significant impact on body composition with increases in total body and thigh lean mass detected in the COPD patients. This is consistent with a previous study reporting a 2.5% increase in thigh muscle mass of healthy elderly individuals who underwent 12 weeks of EET (Mueller et al., 2009). Of additional interest was the decrease in body fat and thigh fat percentage. These findings contrast to those in the CET group where thigh fat and muscle mass were remarkably similar before and after training. Reductions in thigh fat content have been reported in previous studies comparing EET to conventional resistance training in healthy elderly (Mueller et al., 2009, 2011) as well in patients with type 2 diabetes after eccentric resistance combined with aerobic exercise training (Marcus et al., 2008). Although the basis for these observations is currently unclear, eccentric resistance exercise has been reported to alter substrate metabolism in elderly individuals (Krishnan et al., 2003). Further studies into the interesting effects of EET on body composition and lipid metabolism are certainly warranted.

### Eccentric cycling effects on locomotor muscle mitochondria in COPD

In view of the widely reported decline in muscle oxidative capacity in COPD (Remels et al., 2007; Gosker et al., 2007a; Picard et al., 2008; Konokhova et al., 2016) understanding whether a dynamic exercise paradigm emphasizing eccentric muscle actions benefits mitochondrial function is important and relevant for understanding its therapeutic efficacy. However, consistent with our hypothesis, analysis of multiple mitochondrial markers reflecting induction of biogenesis, mitochondrial content, and respiration, revealed no improvements in the locomotor muscle of COPD patients in the EET group. Previous studies assessing the mitochondrial response to EET reported either no change in total mitochondrial volume density or a down-regulation in expression of genes involved in mitochondrial biogenesis (PGC-1α) and function (cytochrome oxidase subunit 4) in healthy young, elderly, and patients with coronary artery disease (LaStayo et al., 2000; Steiner et al., 2004; Zoll et al., 2006; Mueller et al., 2011). Interestingly, in those studies comparing eccentric to concentric cycling modalities where the metabolic exercise intensity was matched, the level was such that it did not induce mitochondrial adaptation in the muscle trained concentrically. In our study, the training intensity (~85% of maximal heart rate) resulted in significant mitochondrial alterations in the group that underwent CET training, where increases in the key mitochondrial biogenesis signaling factor (both PGC-1α mRNA and protein) were detected, along with increases in mitochondrial respiratory chain complex I and complex II content. Furthermore, these increases in mitochondrial biogenesis and content were supported by increases in mitochondrial state 3 and complex IV-driven respiration in the cohort of patients in whom these analyses were made, reflecting the improvement in mitochondrial function after conventional aerobic training. Therefore, our study adds to previous reports describing divergent mitochondrial responses to eccentric cycle training and further emphasizes that even at intensities that impact the mitochondria when exercised concentrically, eccentric cycle training does not induce mitochondrial adaptation. Taken together, findings from these studies highlight the fact that the eccentric modality, even when done in the dynamic rather than resistance mode, is associated with a dominant mechanical stimulus at the expense of metabolic adaptation (Zoll et al., 2006; Mueller et al., 2011).

### Feasibility and therapeutic application of EET in COPD

Given our findings, we believe EET provides a unique strategy in the context of exercise rehabilitation for patients with moderate to severe COPD to increase muscle strength. However, an important consideration in terms of therapeutic application of EET relates to the safety and feasibility of eccentric cycling. The notion that eccentric actions induce muscle damage, injury, and pain likely prevented the application of eccentric exercise as a therapeutic strategy for clinical populations in the past, however more recent studies have shown that a progressive introduction and increase in the intensity, duration and frequency can minimize or avoid these effects (LaStayo et al., 2003). Here we confirm the safety of progressively increasing eccentric cycling intensity over the first 4 weeks, with serum CK levels remaining within normal limits at weeks 2, 5, and 10 of training. Muscle biopsy analysis revealed no significant increases on average in the proportion of central nuclei, further demonstrating that progressive increases in eccentric workload does not induce muscle damage. Furthermore, patients reported EET to be relatively effortless as indicated by their ratings of perceived exertion for both leg fatigue and dyspnea (reaching no higher than a 3 out of 10 on the Borg rating scale). In fact, patients in the EET group reported significantly lower leg fatigue and dyspnea than patients undergoing conventional cycle training at several time points over the 10-week protocol. This is clinically important and has the potential to foster compliance to this strategy in a rehabilitation setting.

EET has been shown to induce numerous functional benefits in other clinical populations (Isner-Horobeti et al., 2013) that can be expected to occur in COPD and warrant future study. For example, eccentric muscle actions have been shown to require distinct neural input (Enoka, 1996) and may in fact improve tasks that involve coordination and movement (Dibble et al., 2006). EET has been shown to improve neural coordination, functional tasks (stair descent, timed up and go), and reduce fall-risk in the healthy elderly (Mueller et al., 2009).

### Study limitations

Despite strengths and unique findings of this study, some limitations should be taken into account. First, our small sample size and inclusion of only male patients with moderate to severe disease may limit the generalizability of our findings to other patients with less advanced COPD as well as female patients. Despite randomization in our study, COPD patients in the EET group had more patients with very severe disease compared to the CET group. In terms of peripheral muscle remodeling, there are conflicting reports that fiber CSA may depend on COPD disease severity (Eliason et al., 2009; Vogiatzis et al., 2011), and that the CSA response to high intensity cycle training may be blunted in patients with severe COPD who have muscle wasting compared to patients with the same disease severity who have normal muscle mass (Vogiatzis et al., 2010, 2011). Clearly, more work is needed to determine what muscle-specific factors influence the variability of training response in severe COPD patients. Relating to this variability in muscle response is the relatively small sample size of patients in our current RCT who underwent muscle biopsies before and after training, as well as the biopsy sampling of muscle tissue at only one site in the thigh. Given the differences in the regional distribution of hypertrophy to eccentric training described previously, sampling at multiple sites along the muscle belly to include the distal and mid portions may have provided more definitive results as to the efficacy of EET on fiber hypertrophy. In addition, measurement of fiber CSA in absolute terms from biopsy material has inherent limitations (Zumstein et al., 1983), and it is possible that the changes in muscle mass and strength after EET may be difficult to resolve in the biopsy samples. Finally, in terms of biopsy-related limitations, we only had sufficient tissue to measure mitochondrial respiration in four patients per group. We believe it is unlikely that this small sample size explains the lack of statistical improvement in EET since we were able to detect significant changes in mitochondrial respiration within the CET group, and thereby interpret the findings to reflect the lack of training adaptation, consistent with the other mitochondrial measures (PGC-1α mRNA and content, electron transport chain complexes) within the EET group showing no improvement. We also did not have enough muscle tissue remaining in all the subjects to examine factors involved in muscle remodeling and repair (extracellular matrix components, satellite cell and growth factors; Mueller et al., 2011) or other mechanical transduction mechanisms that have reportedly been activated through eccentric resistance exercise (Franchi et al., 2014). This would have strengthened the notion of whether the EET was inducing physiological changes within the muscle that could contribute to increases in muscle strength. A final limitation relates to the intensity of the EET. First, the intensity was kept stable after the 4 weeks of training load progression, however several patients expressed interest in wanting to increase their weekly training work rate as it felt relatively easy at later points in the study. This could have influenced the degree of training response that patients experienced, as progressive overload is integral to maintaining continual adaptation of skeletal muscle (Hortobagyi et al., 1996; Lastayo et al., 1999) particularly since the muscle was sampled after 5 weeks of a plateaued workload. Second, while the absolute workload (~150 watts) was three times higher in the EET group compared to CET, higher workloads (~300–340 watts) have been safely applied to healthy octogenerian men and women and patients with coronary artery disease (Steiner et al., 2004; Mueller et al., 2009). The greatest reported magnitude of improvement in muscle strength (36%) and fiber CSA (52%) was achieved when EET workrate was close to 500 watts in a group of healthy participants (LaStayo et al., 2000). Given the safety and tolerability of progressively increasing the eccentric workload documented in our COPD patients, we propose future studies to assess the locomotor muscle response to higher ECC workloads.

### Conclusion and future study

Although the primary lung pathology in COPD is largely irreversible, targeting the adaptability of skeletal muscle through exercise training has therapeutic promise. However, not only are many patients with advanced disease unable to participate in exercise rehabilitation, there is great variability in training responsiveness within the COPD patient population, emphasizing the need to redesign and personalize the exercise rehabilitation approach to optimize therapeutic efficacy (Spruit et al., 2015). Our current study demonstrates the safety and efficacy of EET in patients with moderate to severe COPD, and provides evidence for its inclusion into current pulmonary rehabilitation programs. Our findings reveal improvements in strength, muscle mass, and composition of COPD locomotor muscle in response to EET, with no effect on mitochondrial biogenesis or content. In contrast, mitochondrial adaptations were detected after CET. Based on these divergent findings, we propose the use of dynamic eccentric training to first maximize gains in muscle strength and mass, allowing patients to subsequently undergo conventional cycle training at intensities appropriate to more effectively induce mitochondrial adaptation. High-intensity interval training has been shown to be more potent in stimulating mitochondrial biogenesis and respiration compared to constant load aerobic training (Wisløff et al., 2007; Daussin et al., 2008). While we have shown a robust mitochondrial adaptation to CET in our COPD patients, we believe that the proposed paradigm would allow patients to undergo high-intensity interval training and thus achieve maximal gains in muscle oxidative capacity. Tailoring exercise training modalities to best suit the functional deficits of weakness and fatiguability in the patient are important, and the proposed paradigm warrants consideration.

Further understanding of the activation of molecular and cellular signaling pathways which mediate muscle hypertrophy and nervous system adaptations in response to EET are also needed to better understand this training modality. To confirm the efficacy of EET on increasing muscle mass, future studies need to include serial ultrasound measurements, which have shown good reproducibility and sensitivity to changes in muscle mass in the COPD population compared to DEXA (Menon et al., 2012). Optimizing the EET cycling protocol in terms of absolute workload and progression will be essential in determining the maximal training response. Findings from this study support the inclusion of EET alone or in combination with CET into current pulmonary rehabilitation programs for COPD patients, in order improve skeletal muscle dysfunction, exercise tolerance, quality of life, and reduce mortality risk.

## Ethics statement

This study was carried out in accordance with the recommendations of the Research Institute of the McGill University Health Center. All subjects gave written informed consent in accordance with the Declaration of Helsinki. The protocol was approved by the Biomedical B (BMB) Research Ethics Board, 09-178-BMB.

## Author contributions

Study concept and experimental design: TT, HP, RR, JB; Collection and assembly of data: NM, SK, GG, YK, RdS; Analysis and interpretation of the data: TT, RH, HP, RR, NM, SK, GG, YK; Funding to support study: TT, RH, JB; Provision of study materials or patients: NM, SK, GG, YK; JB, RJ, JB. Administrative, technical, and logistic support: RdS, JB, NM, TC, RA, RJ. Statistical analyses: NM, RdS, TT; Drafting of the manuscript: NM, TT; Critical revision of the article for important intellectual content: NM, TT, RH; RJ; Final approval of the article: all authors read and approved final manuscript.

### Conflict of interest statement

The authors declare that the research was conducted in the absence of any commercial or financial relationships that could be construed as a potential conflict of interest.
