# Supplementary material for: Eccentric Ergometer Training Promotes Locomotor Muscle Strength but Not Mitochondrial Adaptation in Patients with Severe Chronic Obstructive Pulmonary Disease
Source: Front Physiol. 2017 Mar 3;8:114. doi: 10.3389/fphys.2017.00114 (PMC5334343; doi:10.3389/fphys.2017.00114)
Supplement: Supplementary file 1 [file Table1.docx]

**SUPPLEMENTAL**

**Table 1.** Medications taken by COPD patients in each training group.

| **Medication** | **EET (n=8)** | **CET (n=7)** |
| --- | --- | --- |
| Bronchodilators | 8 | 7 |
| Statins | 2 | 3 |
| Anti-arrthymic | 0 | 1 |
| Ca^2+^ channel blockers | 4 | 1 |
| Angiotensin receptor blockers | 0 | 1 |
| ACE inhibitors | 0 | 1 |
| ASA | 5 | 3 |
| Metformin | 1 | 2 |

Data are presented as frequency counts.
