# Supplementary material for: Eccentric Ergometer Training Promotes Locomotor Muscle Strength but Not Mitochondrial Adaptation in Patients with Severe Chronic Obstructive Pulmonary Disease
Source: Front Physiol. 2017 Mar 3;8:114. doi: 10.3389/fphys.2017.00114 (PMC5334343; doi:10.3389/fphys.2017.00114)
Supplement: Supplementary file 2 [file Table2.docx]

**Table 2:** Muscle fiber type distribution before and after training in the EET and CET groups

|  | **EET** | | **CET** | |
| --- | --- | --- | --- | --- |
| **Fiber type** | **Pre** | **Post** | **Pre** | **Post** |
| Type 1 (%) | 19.9 ± 3.3 | 22.3 ± 3.2 | 26.4 ± 5.5 | 28.6 ± 3.3 |
| Type 2a (%) | 47.2 ± 6.1 | 37.6 ± 4.7 | 37.0 ± 6.0 | 38.1 ± 5.4 |
| Type 2x (%) | 0.0 ± 0.0 | 0.1 ± 0.1 | 0.1 ± 0.2 | 0.1 ± 0.2 |
| Co-expressors (%) | 32.9 ± 4.4 | 40.0 ± 4.2 | 36.5 ± 1.8 | 33.2 ± 4.8 |

Data are presented as mean ± SEM. Co-expressors are fibers expressing more than one myosin heavy chain isoform. *Significant difference compared with pre-training values, p < 0.05.
