# Supplementary figures and images for: Eccentric Ergometer Training Promotes Locomotor Muscle Strength but Not Mitochondrial Adaptation in Patients with Severe Chronic Obstructive Pulmonary Disease
Source: Front Physiol. 2017 Mar 3;8:114. doi: 10.3389/fphys.2017.00114 (PMC5334343; doi:10.3389/fphys.2017.00114)

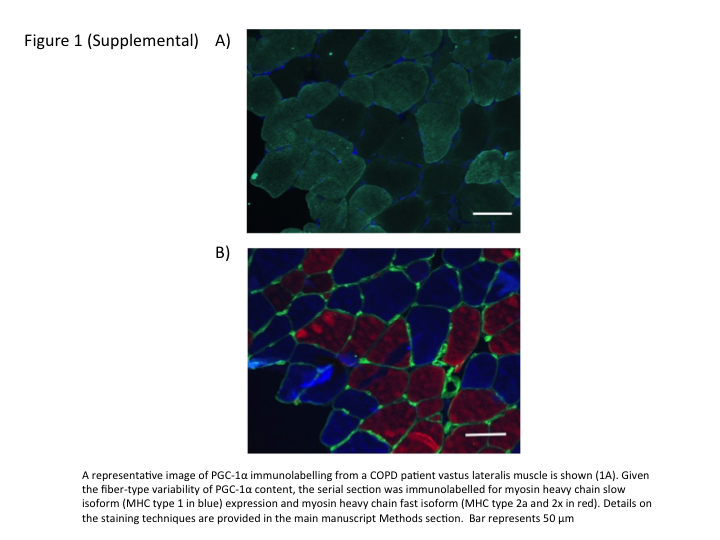

Supplement: Supplementary file 3 [file Image1.TIFF]
